# Evaluation of contrast Sonazoid-enhanced ultrasonography for the detection of hepatic metastases in breast cancer

**DOI:** 10.1007/s12282-014-0560-0

**Published:** 2014-08-21

**Authors:** Mai Mishima, Uhi Toh, Nobutaka Iwakuma, Miki Takenaka, Mina Furukawa, Yoshito Akagi

**Affiliations:** Department of Surgery, Kurume University School of Medicine, 67 Asahi-machi, 830-0011 Kurume, Fukuoka Japan

**Keywords:** Breast cancer, Liver metastasis, B-mode ultrasonography, Contrast Sonazoid-enhanced ultrasonography

## Abstract

**Background:**

The present study was aimed to evaluate the usefulness of contrast Sonazoid-enhanced ultrasonography (US) for the detection of hepatic metastases in breast cancer patients and compare the clinical efficacy and sensitivity of this technique with conventional contrast unenhanced B-mode US in follow-up examinations of breast cancer patients with liver metastasis.

**Methods:**

We assessed a total of 84 hepatic tumors from 24 patients diagnosed with or suspected of having metastatic cancer. These hepatic nodules were diagnosed through imaging, including dynamic magnetic resonance imaging (MRI), contrast-enhanced computed tomography (CECT) scan, B-mode US or contrast Sonazoid-enhanced US (SEUS). Differences in the sensitivity between US and SEUS were compared using MR imaging, CECT, and follow-up imaging.

**Results:**

A total of 79 nodules were diagnosed as metastatic tumors. The remaining nodules were diagnosed as benign tumors (hepatic hemangioma: *n* = 3; local fatty change: *n* = 2). SEUS precisely detected the presence or absence of hepatic tumors in the 24 patients examined, showing a sensitivity of 98.8 % (83 of 84 lesions) for total imaged solid liver lesions, with an accuracy of 98.7 % (78 of 79 lesions) for total metastatic breast cancer lesions. In contrast, conventional B-mode US imaging revealed hepatic tumor lesions at a sensitivity of 66.7 % (56 of 84 lesions) and an accuracy of 64.6 % (51 of 79 lesions), respectively. Furthermore, the false positive and false negative rates were, respectively, 6.33 and 29.1 % for B-mode US and 0 and 1.3 % for SEUS. Moreover, twenty-seven metastatic tumors and five benign lesions (3 hemangiomas and 2 focal fatty changes/sparings) were imaged using SEUS but not conventional B-mode US. Significant differences in diagnostic accuracy rates between contrast Sonazoid-enhanced US and conventional B-mode US were observed (Wilcoxon signed rank test: *p* = 0.0009). No severe adverse events occurred during SEUS after the administration of Sonazoid, except for a grade 1 skin reaction and nausea in one patient.

**Conclusion:**

These results suggested that Sonazoid could be safely administrated to breast cancer patients with liver metastatic disease. Thus, contrast Sonazoid-enhanced US is a feasible and more effective method than B-mode US for the detection of hepatic metastasis, particularly for small metastatic breast cancer lesions less than 14 mm in diameter, showing significant high sensitivity and accuracy.

## Introduction

Hepatic metastasis is a major problem in breast cancer patients. The detection of these lesions has treatment and prognostic implications, and accurate staging is also a prerequisite for monitoring chemotherapy. Although an abdominal liver ultrasound is not recommended for routine breast cancer surveillance, as to other conventional examinations, ultrasonography has been reported as effective for the “early” detection of hepatic recurrences [1, 2]. Despite the recent development of new techniques, including 18-fluro-2-deoxyglucose (FDG) positron emission tomography (PET) computed tomography (CT) and dynamic magnetic resonance imaging (MRI), for the detection of hepatic metastasis from breast cancer, the use of ultrasonography (US) for the assessment and follow-up of hepatic metastatic disease might benefit from a reliable, easily available, low cost, noninvasive imaging modality. Although B-mode US is commonly performed as a screening examination, most of the conventional US techniques have relatively poor sensitivity and specificity for imaging liver metastases (53–76 %), and trans-abdominal US is inferior in sensitivity for liver metastases compared with CT or MRI primarily reflecting a lack of contrast agents. Currently CECT and MRI are the only imaging modalities that offer the highest diagnostic potential for the assessment of liver metastases in breast cancer patients [3, 4].

Contrast-enhanced US has been demonstrated as a suitable technique for the detection of hepatic malignancy or metastases and is more accurate compared with conventional B-mode US [5–9]. However, the efficacy of contrast-enhanced US for the detection of hepatic metastases from breast cancer has not been specifically evaluated. Sonazoid (Diichi-Sankyo, Tokyo, Japan) is a new microbubble agent that provides a parenchyma-specific contrast image based on its accumulation in the Kupffer cells of the liver [10–14]. Sonazoid has previously been approved for clinical use in Japan, and this agent presents a image in the post-vascular phase (Kupffer image) with a long duration, followed by the images of the arterial phase and the portal phase (vascular phase) [15, 16]. In the present clinical study, we demonstrate the use of Sonazoid in contrast-enhanced US for the detection of hepatic metastasis in patients with breast cancer.

## Patients and methods

### Patients

Between September 2011 and December 2012, 24 female patients (median age 59 years, range 41–72 years) with histologically diagnosed metastatic breast cancer were included in this study. Inclusion criteria were referral to the radiology department for CECT and/or US of the liver for suspected or known hepatic metastases. In addition to the hepatic metastases from primary breast cancer, the other metastatic sites included bone (*n* = 17), lung (*n* = 8), distant lymph nodes (*n* = 5), brain (*n* = 3) and skin–chest wall recurrence (*n* = 2). The median number of sites of metastatic disease before the entry was 3 (range 1–5sites). All patients with metastatic disease were treated with conventional chemotherapy and/or endocrine therapy using weekly paclitaxel with trastuzumab for 3 patients, triweekly docetaxel with trastuzumab for 4 patients, Eribulin for 2 patients and Capecitabine for 1 patient. The remaining 14 patients underwent endocrine therapy, including Tamoxifen for 4 patients and aromatase inhibitor for 10 patients (anastrozoles for 5 patients, letrozoles for 3 patients, and exemestanes for 2 patients). In addition, 3 patients with Her-2 positive metastatic disease also underwent concurrent trastuzumab treatment (Table 1). All patients provided written informed consent for participation in this study.

**Table 1:** Characteristics of the patients with suspected hepatic metastases and adverse events during SEUS

Characteristics ^a^	No.	Percentage
Age (median)	59 years old (41–72)	
Menopausal status		
Premenopause	4	17 %
Postmenopause	20	83 %
Intrinsic subtype		
Luminal phenotype	9	38 %
HER2 phenotype	9	38 %
Triple negative phenotype	6	25 %
Sites of metastases beside liver		
Bone	17	
Lung	8	
Distant lymph nodes	5	
Brain	3	
Skin-chest wall recurrence	2	
Treatment^b^		
Chemotherapy (+ trastuzumab)	10 (6)	42 %
Endocrine therapy (+ trastuzumab)	14 (3)	58 %
Adverse event during SEUS	No.	Grade
Nausea	1	1
Vomiting	0	
Diarrhea	0	
Edema	0	
Itching	1	1
Skin rush	0	
Headache	0	0

^a^The median follow-up time was 14.1 months (range 9–28 months)

^b^The treatment for metastatic disease included chemotherapy with trastuzumab for 6 patients (3 patients were treated weekly with Paclitaxel and 4 patients were treated triweekly with docetaxel), endocrine therapy with trastuzumab for 3 patients, Eribulin was administered to 2 patients and Capecitabine was used to treat 1 patient. The remaining eleven patients underwent endocrine therapy only

### Imaging techniques, equipments and imaging assessment

**Fig. 1:**
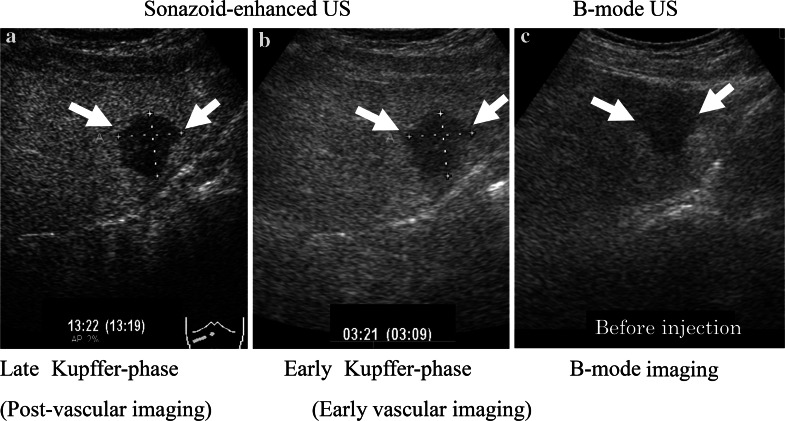
More hepatic metastases (*arrows*) were identified as perfusion defects (**a**) in the post-vascular phase (Kupffer phase) than at the early vascular phase (**b**) or before the administration of Sonazoid using conventional B-mode US (**c**)

All patients underwent conventional US, contrast Sonazoid-enhanced US (SEUS), and CECT or MRI. The metastatic nature of the liver lesions was determined on the basis of progression or remission through imaging after chemotherapy or endocrine therapy. Four experienced radiologists, blinded to any other imaging data, performed the B-mode and SEUS scanning using an Aplio-400 (Toshiba, Tokyo, Japan). The Aplio-400 PVT-375BT Transducer/Probe had been used with a 3.5 MHz center frequency. Adverse events occurring up to 24 h post injection were recorded according to Common Terminology Criteria for Adverse Events (CTCAE version 4.0). The patients received a bolus intravenous injection of Sonazoid (0.015 mL/kg body weight) through a peripheral venous line, followed by 10 mL of normal saline flush. After the administration of Sonazoid, the portal veins, hepatic veins, and normal liver parenchyma were uniformly enhanced immediately (vascular phase image). Approximately 10 min after the injection, the liver was scanned again to observe the post-vascular image (Kupffer image). The hepatic metastases were identified as perfusion defects (Fig. 1a) clearly more visible in the post-vascular image than those in the B-mode US (Fig. 1c) or early vascular phase (Fig. 1b) including images of the arterial phase and the portal phase, which were captured less than 10 min after Sonazoid injection. The post-vascular phase image (Kupffer image) lasted at least for 20 min.

**Fig. 2:**
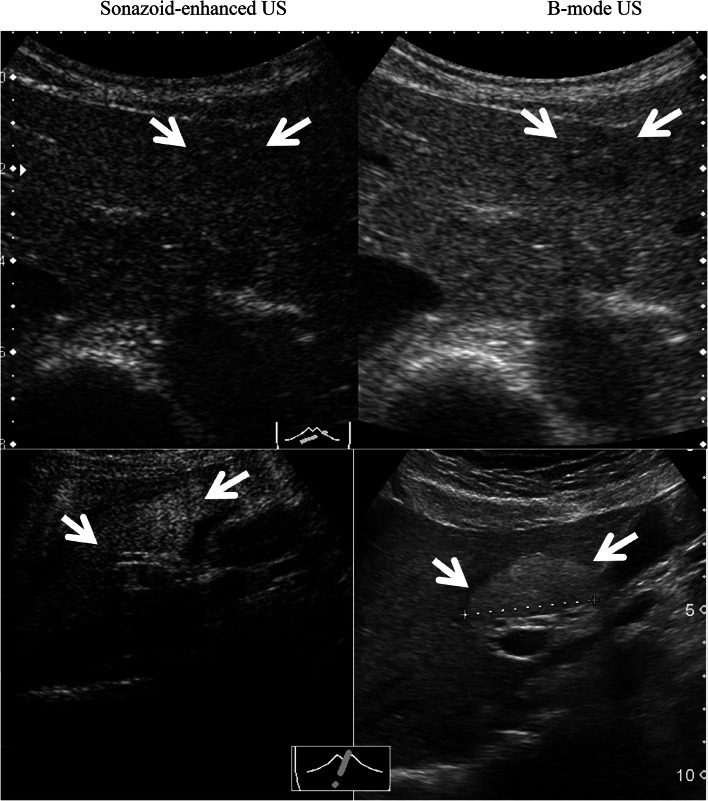
In SEUS, lesions showing iso-enhancement as a hemangioma (*upper*) and iso- or hyper-enhancement as a focal fatty change/sparing (*below*) were considered benign (*arrows*)

A total of 24 patients underwent SEUS in addition to B-mode US. The number and size of the metastases identified through CECT and/or MR were compared with those detected using B-mode US and SEUS. In SEUS, if a lesion showed reduced enhancement (defect/washout), then it was considered metastatic (Fig. 1) and if it showed iso- or hyper-enhancement, then the lesion was considered benign (Fig. 2), compared with the surrounding liver in the post-vascular phase. Hepatic metastases were identified in most cases as perfusion defects in the post-vascular phase image, and this image lasted 10–30 min after the injection of Sonazoid, and in some cases, showed hypoechoic changes with rim enhancement, which also distinguished metastases from most other masses. Based on baseline ultrasonography, metastases were defined as clearly visible round, oval, or lobulated solid focal lesions that were neither simple cysts nor typical of hemangioma, focal fatty sparing or change. In SEUS, metastases were defined as sharply marginated round, oval, or lobulated hypoechoic defects in enhancing parenchyma in the portal venous or post-vascular phase (Kupffer phase). Further, tumor vessels and tumor enhancement of liver metastases were visualized immediately in the early vascular phase (arterial phase) following Sonazoid administration. However, not only relatively large vessels including tumor vessels and portal veins, but also microvessels within the liver parenchyma are rapidly fulfilled with Sonazoid microbubbles, which in turn permit rapid enhancement of the tumor and liver parenchyma simultaneously. For each patient, the final number of hepatic metastases present at the time of US and SEUS was determined based on a consensus reading, including the results of MR imaging examination or CECT, US, SEUS, and follow-up imaging examinations.

### Statistics analysis

The standard was determined as the number of metastases revealed through a combination of CECT or MRI and follow-up. The sensitivity and accuracy of both types of US for metastases were calculated in the detection of individual metastases for each patient, and the sensitivities were compared using the Wilcoxon signed rank test. A *p* value of less than 0.05 was considered statistically significant.

## Results

### Clinical results

All twenty-four patients with suspected hepatic metastatic tumors (Table 1) received at least twice Sonazoid injections in each examination, and no general or cardiovascular complications or severe adverse events were observed during the procedure. Only one patient complained of mild nausea (Grade 1) and a skin reaction (Grade1) on the injected site after Sonazoid administration.

Follow-up examinations were performed every 3 months, and equivocal lesions under B-mode US and SEUS were clarified through computed tomography (CT) scan and/or magnetic resonance imaging (MRI). The median follow-up was 14.1 months (range 9–28.2 months).

**Fig. 3:**
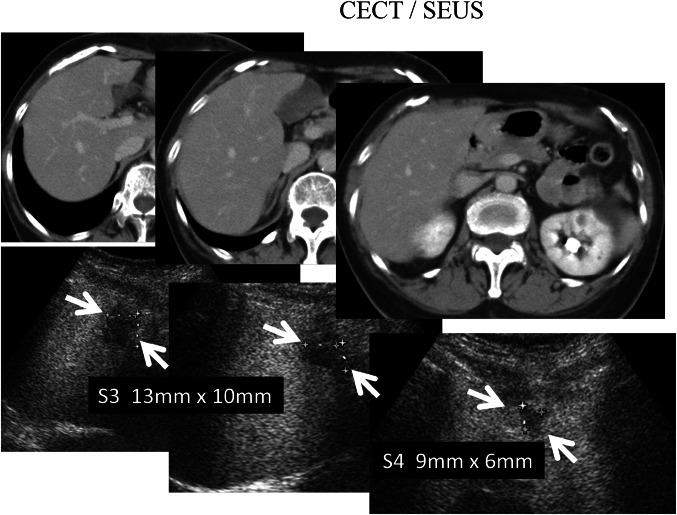
Four lesions were observed with SEUS (*below arrows*) but not with CECT (*upper*). Three of the four lesions were shown to be small metastases (4–13 mm at the time of the diagnosis) during follow-up imaging, reflecting the increase in lesion size

The total number of suspected metastatic lesions upon initial examination was 84. A total of 19 patients presented hepatic metastases, detected as a total of 79 metastatic lesions using contrast-enhanced MRI and/or CECT; no metastases were observed in 5 patients. A total of 74 hepatic metastases were observed in 19 patients using CECT. Four lesions were observed using SEUS, but not with CECT; these lesions were revealed as small metastases (4–14 mm at the time of the diagnosis) upon follow-up imaging, showing an increase in lesion size (Fig. 3). There was no exclusion based on poor ultrasonographic conditions, and the population included a total of 79 hepatic metastases in 19 patients. SEUS revealed 78 out of 79 metastases with tumor sizes ranging from 4 to 174 mm in diameter. One lesion was missed using both US and SEUS detection methods compared with the MRI findings; this subdiaphragmatic lesion was not accessible to ultrasonography. SEUS showed more MRI-confirmed metastatic lesions, detecting 83 of the 84 lesions with 98.8 % sensitivity, compared with conventional US, which only detected 56 lesions in 11 of the 19 patients, with 66.7 % sensitivity. SEUS and conventional US revealed suspected malignant breast cancer metastases in a total of 78 and 51 lesions, respectively, and the true positive rates (accuracy) were 98.7 % (78/79) and 64.6 % (51/79), respectively (Wilcoxon signed rank test *p* = 0.0009) (Table 2).

**Table 2:** Results of Sonazoid-enhanced ultrasonography for patients with hepatic metastasis of breast cancer

	B-mode	Sonazoid	CECT	MRI	B-mode	Sonazoid
					No. false positive cases	
Total lesions of suspected metastases^a^	84	84	84	84		
Total detected lesion	56	83	80	84		
Total lesions of MBC^b^	79	79	79	79		
Detected No. MBC	51	78	75	79		
Detected No. other diseases					5	0
Hemangioma	0	3	3	3	3	0
Focal fatty change	0	2	2	2	2	0
Sensitivity (%)^c^	66.7	98.8	95.2	100		
Accuracy (%)^c^	64.6	98.7	94.9	100		
Positive predictive rate (%)	70.9	98.7				
False positive rate (%)					6.3	0
False negative rate (%)	29.1	1.3				

^a^24 patients with total 84 hepatic tumors having or suspected of having metastatic cancer from breast cancer

^b^19 patients with a total 79 metastatic breast cancer (MBC) lesions, diagnosed through contrast-enhanced MRI and/or CECT

^c^Statistical significance

**Fig. 4:**
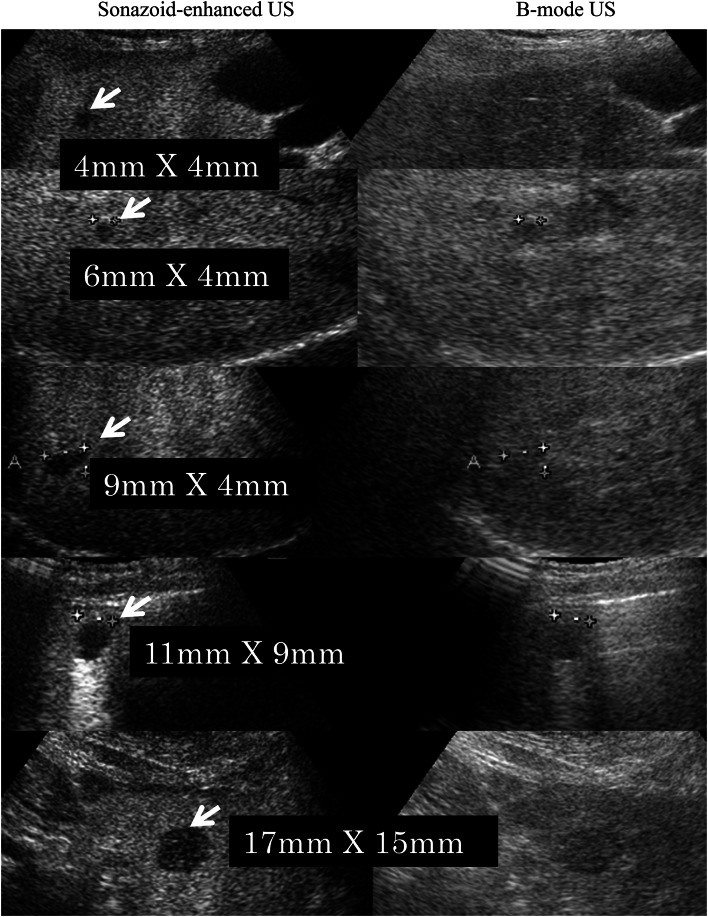
The lesions missed with US and detected with SEUS (*white arrows*) ranged in size from 4 to 17 mm, with an average maximum diameter of 14 mm

**Fig. 5:**
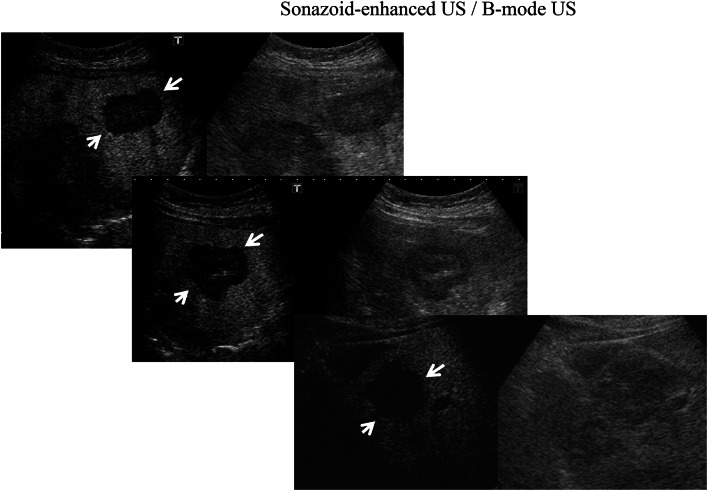
Approximately 82.3 % of the liver metastases of breast cancer showed hypoenhancing or filling defects (*white arrows*) in the post-vascular phase

**Fig. 6:**
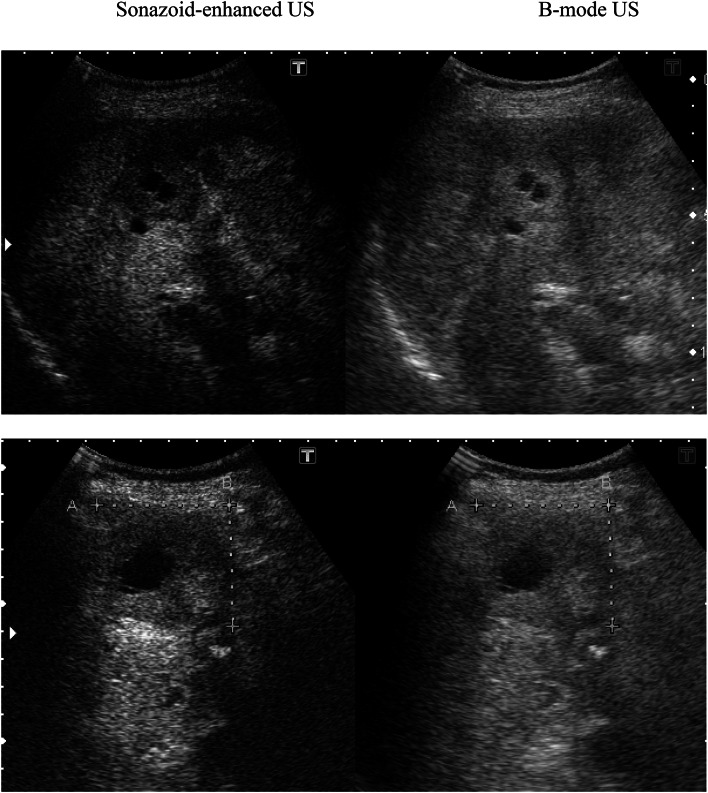
Approximately 17.7 % of the liver metastases of breast cancer displayed hypoechoic changes with rim enhancement in the post-vascular phase

The mean maximum diameter of the lesions was 32 mm (range 4–174 mm). The lesions missed using B-mode US and detected with SEUS had a median maximum diameter of 14 mm, ranging from 4 to 17 mm (Fig. 4). The average sensitivity for the detection of individual metastases improved significantly from 66.7 to 98.8 % (Wilcoxon signed rank test *p* = 0.0112). False-positive results were identified in five lesions in five different patients using conventional B-mode US; three lesions were hepatic hemangiomas and two lesions were focal fatty change/sparing (Fig. 2), diagnosed through SEUS, CECT and confirmed upon follow-up imaging. No false-positives results were observed with SEUS. However, four lesions were missed under CECT scan examination and detected only through SEUS, likely reflecting the small size of the lesion, with a diameter of less than 13 mm (Fig. 3). Only one patient, showing false negative rates using SEUS (also on B-mode US), presented a subdiaphragmatic lesion that was not typically accessible to ultrasonography. Large metastatic lesions were markedly enhanced in the post-vascular phase (Kupffer phase), either homogeneously or peripherally. In the post-vascular phase, the liver metastases in breast cancer patients showed hypoenhancing or perfusion defects in 65 of 79 lesions (82.3 %) (Fig. 5), in contrast, fourteen of the 79 lesions (17.7 %) displayed hypoechoic changes with rim enhancement (Fig. 6).

## Discussion

In the present study, we demonstrated that liver metastasis from breast cancer frequently shows hypoechoic defects under contrast Sonazoid-enhanced US compared with the surrounding normal liver parenchyma enhanced through increased echogenicity resulting from treatment with a Sonazoid microbubble contrast enhancer (Fig. 1).

Small liver metastases from breast cancer were only detected through contrast Sonazoid-enhanced US, and conventional US did not show abnormality in metastatic lesions ranging from 4 to 17 mm in diameter. Similarly, CECT did not detect micrometastatic lesions of less than 13 mm in diameter. In contrast, SEUS at the post-vascular phase (Kupffer imaging) revealed 4–17 mm rounded small hepatic metastatic lesions (Figs. 3 and 4), compared with CECT scan showed a low sensitivity for detection and characterization of lesions smaller than 1 cm [17]; contrast-enhanced US had detected small hepatic metastatic lesions (<1 cm) with a high sensitivity [18]; and in this study, four additional small lesions had been revealed by SEUS, but not by CECT. These results suggest SEUS has better contrast resolution than CECT for detecting small hepatic metastatic lesions, and SEUS may improve the detection of miliary metastases (0.5–1 cm) [19].

The detection of large liver metastases from breast cancer using conventional B-mode US and SEUS in the post-vascular phase, revealed that 82.3 % of most enhanced lesions showed reduced enhancement (defect/washout) (Fig. 5), and 17.7 % of the lesions showed hypoechoic changes with rim enhancement in the post-vascular phase using SEUS, which was not visible through baseline ultrasound (Fig. 6).

In the present study, contrast Sonazoid-enhanced US showed higher sensitivity and accuracy for the detection of liver metastases from breast cancer compared with conventional unenhanced B-mode US. Several clinical trials involving contrast-enhanced US using Sonazoid to detect hepatic metastasis have been performed worldwide in various cancers [9, 20–22] and these previous studies have also demonstrated the improvement in accuracy of contrast-enhanced US in diagnosing hepatic metastasis [23–25]. Differential diagnosis of hepatic metastasis between breast cancer and other original cancer may indeed be a problem, there were some reports demonstrating that hepatic cellular carcinoma shows increased enhancement in the arterial phase, metastasis from gastrointestinal cancer and neuroendocrine tumor can be identified with the hypoenhancement in the portal venous and the post-vascular phase [22]. However, as far as we know, no published study has specifically reported the value of SEUS for detecting hepatic liver metastases from breast cancers.

Contrast-enhanced ultrasound with Sonazoid detected significantly more metastases compared with conventional US, with a sensitivity of 98.8 versus 66.7 %. However, SEUS revealed no additional patients with metastatic disease. The hepatic metastases detected through SEUS were indeed relatively small lesions (4–17 mm), often associated with larger lesions. The detection of hepatic metastases from breast cancer using conventional US is limited by the relatively small difference in background patterns between the lesions and hepatic parenchyma, resulting in poor contrast differentiation between the two tissues, likely reflecting the difficulty in definitively diagnosing liver hemangioma and focal fatty change/sparing lesions using only B-mode US. The use of an ultrasound contrast agent such as Sonazoid increases the echogenicity of the liver at the post-vascular specific phase as the microbubbles accumulate in the normal parenchyma.

Although we did not specifically compare the value of the different vascular phases in the detection of metastases from breast and other cancers, the post-vascular phase image (Kupffer image) was valuable, as 83.3 % of the metastases from breast cancer were perfusion defects in the parenchyma, and 17.7 % of breast cancer metastases was iso- or hypoechoic compared with liver parenchyma, showing hypoechoic changes with rim enhancement in the post-vascular phase. However, we did not confirm this finding quantitatively because only 14 of the 79 lesions detected showed central necrotic hypoechoic changes. Notably, the internal content of the metastatic lesions or the areas showing necrotic changes were also correctly identified using SEUS. Because the data from the 19 patients subjected to CECT was limited, the difference between SEUS and CECT scan imaging was not significant in this study. Four lesions were missed by dynamic CT scan examination, but these lesions were identified using SEUS, likely reflecting the small size of the lesion. However, one patient, who showed false-negative results after both baseline B-mode and SEUS, had a subdiaphragmatic lesion that was not accessible to sonography. The limitations of dynamic CT scanning make it difficult to detect small metastases, and the limitations of US make it difficult to visualize subdiaphragmatic lesions.

Furthermore, ultrasound contrast agents, including Sonazoid modify the basic physical interactions between ultrasound waves and hepatic tissues and amplify the signal produced by flowing blood. Thus, Sonazoid might be useful for detecting subtle flow abnormalities and distinguishing areas of abnormal flow relative to normal background parenchymal perfusion. As a result, these contrast agents might improve the characterization of focal liver lesions compared with standard B-mode US, while providing complementary information with other imaging modalities [26–28]. In the present study, we also demonstrated using contrast Sonazoid-enhanced US for the adequate detection of benign lesions, including three hepatic hemangiomas and two focal fatty changes, in patients with metastatic breast cancer.

Thus, these results suggest that SEUS, followed by conventional B-mode US for evaluating breast cancer metastases, might not only be used to successfully detect malignant lesions with higher sensitivity and accuracy, but also to identify benign lesions, including hepatic hemangioma or focal fatty changes, for the differential diagnosis of hepatic lesions. Moreover, because it is competitive, cost effective and less invasive, SEUS technique used for follow-up of hepatic metastases from breast cancer may be an alternative to other imaging modalities including CT scan and MRI. Consequently, the development of a new SEUS approach could improve the diagnostic sensitivity and detection accuracy for hepatic breast cancer metastases and could also provide important information for making treatment decisions for patients with breast cancer.

These results were presented at the 72nd Annual Meeting of the Japanese Breast Cancer Society in June 2013.
